# Understanding Health Inequality, Disparity and Inequity in Africa: A Rapid Review of Concepts, Root Causes, and Strategic Solutions

**DOI:** 10.1002/puh2.70040

**Published:** 2025-03-11

**Authors:** Gabriel Ilerioluwa Oke, Olivier Sibomana

**Affiliations:** ^1^ University of Global Health Equity Butaro Rwanda; ^2^ Department of General Medicine and Surgery College of Medicine and Health Sciences University of Rwanda Kigali Rwanda

**Keywords:** Africa, disparity, healthcare, inequality, inequity

## Abstract

**Introduction:**

Health inequality, disparity, and inequity are critical global health challenges, particularly in low‐ and middle‐income countries (LMICs). Africa's healthcare systems have been profoundly impacted by these issues for decades, leading to severe and far‐reaching consequences. This rapid review aims to explore the concepts of health inequality, disparity, and inequity in Africa, identify their root causes, and propose practical recommendations to address them.

**Methodology:**

A comprehensive search was conducted across PubMed, Google Scholar, the Directory of Open Access Journals (DOAJ), and through manual searches on Google to identify literature on health inequalities, disparities, and inequities in Africa. Key terms, such as “Health inequality,” “Health disparity,” “Health inequity,” “Health injustice,” “Health unfairness,” “Healthcare access,” and “Health challenges,” were employed. The identified literature was carefully reviewed, and relevant sources were selected to discuss the concepts, causes, and potential solutions to these differences in health outcomes across Africa.

**Results:**

Although some aspects of health inequality are linked to unmodifiable factors, health disparities and inequities are often the result of preventable and modifiable factors and can be addressed through targeted efforts. The root causes of health inequality, disparity, and inequity in Africa include poverty, inadequate education, corruption, poor governance, geographic isolation, unsupportive environmental conditions, cultural norms, and international influences. Solutions to these differences in health outcome lie in reversing the root causes.

**Conclusion:**

Addressing health inequality, disparity, and inequity in Africa requires a multifaceted approach, including improving education, healthcare infrastructure, and gender equality, alleviating poverty, and ensuring fair governance to achieve equitable health outcomes.

## Graphical abstract

This rapid review examines health inequality, disparity, and inequity in Africa, identifying poverty, inadequate education, poor governance, and cultural norms as key drivers. Addressing these challenges through improved healthcare infrastructure, education, gender equality, and equitable policies is essential for achieving fair and sustainable health outcomes across the continent.

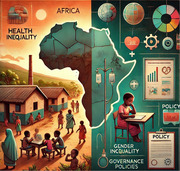

## 1 Introduction

Health inequality, disparity, and inequity are critical global health issues, particularly in low‐ and middle‐income countries (LMICs). Due to socioeconomic, geographic, and political reasons, these regions have notable differences in healthcare outcomes, quality of care, and access [1]. Particularly, noticeable health inequities affect underprivileged groups, who face avoidable and unfair health disparities as a result of structural obstacles, including poverty, poor infrastructure, and restricted educational opportunities [2]. In LMICs, these disparities lead to increased incidence of infectious diseases, noncommunicable diseases, and maternal and infant mortality [3]. Resolving these issues calls for all‐encompassing approaches, such as community‐based treatments, fair resource allocation, and regulatory changes, to guarantee that everyone, regardless of socioeconomic background, can attain ideal health outcomes.

Health inequality, disparity, and inequity, though related, represent distinct concepts in public health discourse. According to Lee et al., health inequality refers to variations in health outcomes across different population groups, whereas health disparities are systematic differences in these outcomes, often caused by the unequal distribution of social conditions, leading to measurable variations in health. On the other hand, health inequity goes a step further, highlighting disparities that stem from avoidable and unjust differences in social, economic, geographical, or healthcare resources [4]. Health inequities are particularly concerning because they arise from unfair conditions that could be addressed, thus reflecting broader issues of social justice and access [5]. The COVID‐19 pandemic highlighted how disadvantaged populations face disproportionate health effects, with minorities experiencing higher mortality rates [6]. These inequalities are often rooted in social factors, creating health disparities among different groups and regions [7]. Efforts to reduce these disparities involve understanding the drivers of these differences, implementing evidence‐based policies, and promoting access to essential resources like healthcare, education, and employment [8].

Africa has markedly health disparities, with large differences in health outcomes and access to care between regions, socioeconomic classes, and genders. There are often significant disparities in life expectancy and disease prevalence between urban and rural areas due to the superior healthcare infrastructure and services provided to only urban communities [9]. Socioeconomic differences make these inequities even worse as they allow wealthier people to have greater access to healthcare and better health outcomes than they do for poorer people [10]. Furthermore, women and girls are more susceptible to poor health outcomes due to gender disparities, which are fueled by systematic discrimination and cultural norms, particularly in areas like reproductive health [11]. Addressing these challenges necessitates a multidimensional approach that prioritizes strengthening healthcare systems, addressing social determinants of health, and implementing equity‐focused policies to ensure universal and equitable access to medical resources.

As health inequalities, disparities, and inequities are obvious in Africa, it is essential to comprehend them completely, along with their underlying causes, in order to develop and implement solutions to deal with them. Developing focused solutions requires a deep understanding of these discrepancies and their contributing factors, including the socioeconomic, geographic, and gender‐based factors. Comprehending these underlying factors can be beneficial in formulating strategies and initiatives that specifically tackle the obstacles impeding fair and equal healthcare accessibility. This review seeks to shed light on the health inequalities, disparities, and inequities in Africa, with a particular emphasis workable solution that may be put into place to help address these problems.

### 1.1 Methodology

A comprehensive search was conducted using PubMed, Google Scholar, and the Directory of Open Access Journals (DOAJ) to identify literature on health inequalities, disparities, and inequities in Africa. Key terms, such as “Health inequality,” “Health disparity,” “Health inequity,” “Health injustice,” “Health unfairness,” “Healthcare access,” and “Health challenges,” were employed to ensure a broad and thorough search. Additionally, a manual Google search was performed to locate institutional, organizational, and government reports addressing health inequality, disparity, or inequity issues and their solutions within the African context. The identified literature was carefully examined, and only relevant sources were included in this rapid review. The aim was to understand the concepts, identify root causes, and highlight interventions to tackle health inequalities, disparities, and inequities across Africa.

## 2 Results and Discussion

### 2.1 Understanding the Concept of Health Inequality in Africa

Even though health inequality can be used to mean disparity or differences in health outcomes in general, it is actually referred to as differences in health status and outcomes among particular groups, which are influenced by genetic or other factors that are beyond prevention or modification [4]. The factors contributing to health inequalities are not unique to Africa; they are almost all applied across diverse global populations. Although many of these determinants are non‐modifiable, this does not imply that health inequalities are insurmountable. Effective interventions can still be implemented to address these inequalities. Using the examples of malaria in individuals with sickle cell disease and incidence of cancers by gender can effectively illustrate the concept of health inequality and how it can be addressed in Africa.

Sickle cell disease comprises a set of inherited disorders affecting red blood cells and their ability to carry oxygen due to abnormalities in hemoglobin. Typically, red blood cells are disc‐shaped and flexible, allowing them to pass smoothly through blood vessels. However, in sickle cell disease, a genetic mutation causes the cells to take on a crescent or “sickle” shape. These misshapen cells lack flexibility and can obstruct blood flow, impeding the delivery of oxygen throughout the body [12]. Sickle cell disease reduces malaria susceptibility due to the protective effect of the sickle cell trait: The mutation in the hemoglobin gene causes red blood cells to assume a sickle shape under low oxygen conditions, which makes it difficult for the malaria parasite Plasmodium falciparum to thrive and complete its life cycle inside these cells [13]. In Cameroon, Eleonore et al. found that malaria affected 23.5% of patients with sickle cell disease, compared to 44.9% of individuals without the condition [14]. In Uganda, malaria prevalence was 33% versus 78% in children with and without sickle cell disease [15]. The reduced susceptibility to malaria among individuals with sickle cell disease is a phenomenon observed globally but is particularly notable in regions where malaria is endemic, such as sub‐Saharan Africa, and is a good example in explaining health inequality in Africa.

**FIGURE 1:**
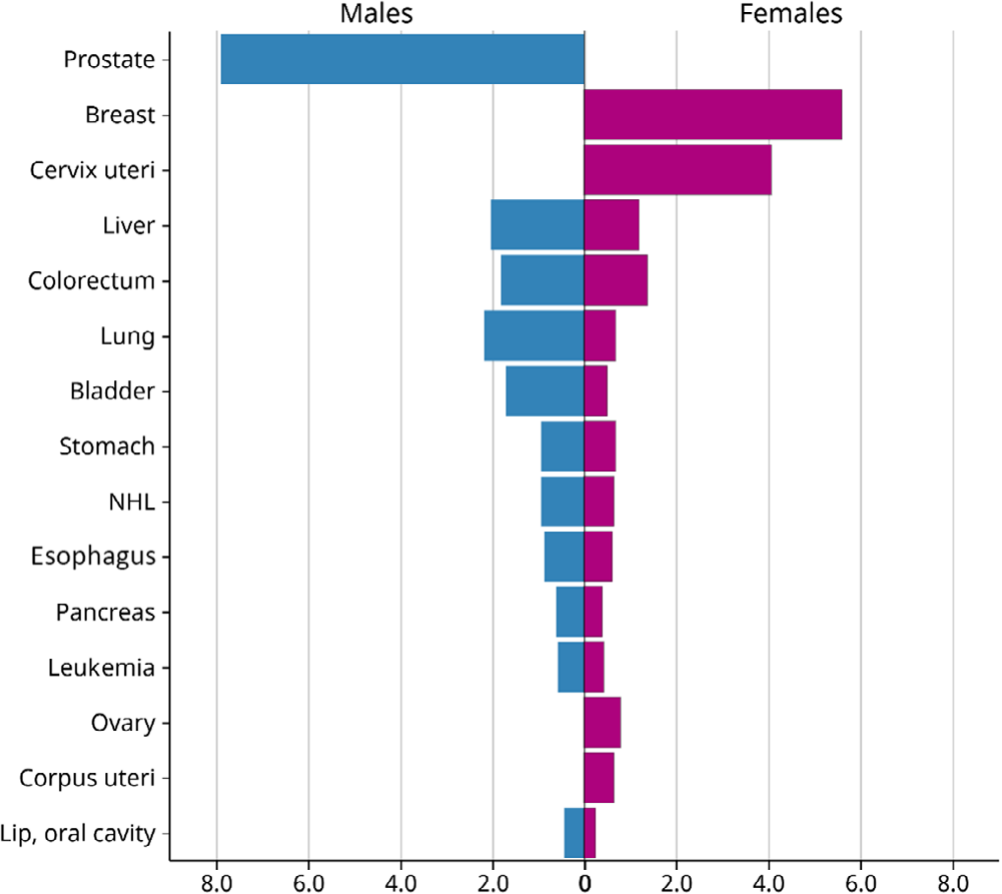
Gender inequalities by type of cancer: age standardized incidence rate per 100,000 individuals of Top 15 cancers in Africa, 2022. *Source:* International Agency for Research on Cancer; https://gco.iarc.fr/en.

Figure 1 illustrates gender inequality in the incidence of the Top 15 cancers within the African population in 2022. Although many cancer types, such as stomach, pancreas, liver, and colorectal cancer, are prevalent in both genders, some cancers show a distinctly unequal distribution. Breast cancer, cervical cancer, corpus uteri cancer, and ovarian cancer exclusively affect females, whereas prostate cancer is limited to males. This observed inequality in cancer distribution is attributed to inherent biological differences, as organs like the prostate are found only in males, whereas the cervix and ovaries are exclusive to females. These differences highlight the influence of gender‐specific anatomical and physiological factors on cancer incidence, which in turn explains the concept of health inequality.

### 2.2 Understanding the Concept of Health Disparity in Africa

Health disparities refer to variations in health outcomes among different population groups. Unlike health inequalities, which encompass all differences in health status, health disparities specifically arise from potentially avoidable health differences that disproportionately affect socially disadvantaged groups. These disparities are often the result of systemic factors, such as unequal access to healthcare, socioeconomic status, and environmental conditions, which lead to adverse health outcomes in these populations [16].

**FIGURE 2:**
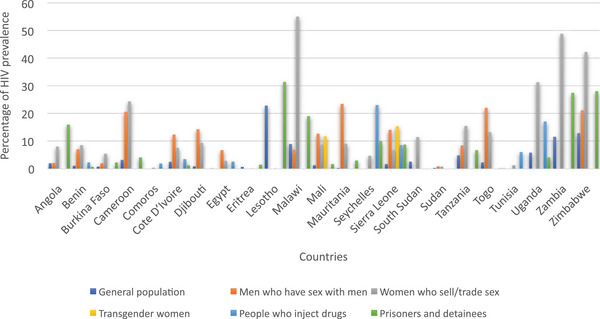
Disparities in prevalence of HIV by population groups in Africa. *Source:* UNAIDS 2020 Global AIDS Update.

HIV prevalence is markedly higher among certain key populations compared to the general population. In Sierra Leone, for instance, the prevalence of HIV in 2020 was 1.6% among the general population. However, specific groups exhibited significantly higher rates: 14% among men who have sex with men, 6.7% among women who sell or trade sex, 15.3% among transgender women, 8.5% among people who inject drugs, and 8.7% among prisoners and detainees. The trend is also the same in other African countries (Figure 2). Giving an example on sex workers, they exhibit a higher prevalence of HIV compared to the general population due to several risk factors associated with their profession, including the frequent engagement in multiple sexual partnerships and a higher incidence of unprotected sex which significantly elevates their vulnerability to HIV infection. This is a good example of health disparity as the contributing factors are avoidable and can be prevented.

### 2.3 Understanding the Concept of Health Inequity in Africa

Health inequities are differences in health outcomes among groups of people that are caused by social, economic, demographic, or geographic injustice and unfairness. The case of Ogoni people in Nigeria can help to explain well what health inequity is. The Ogoni are an indigenous ethno‐linguistic group in the Niger Delta of South‐Eastern Nigeria, constituting about 1% of the Nigerian population. Since Nigeria's independence, they have faced systemic political marginalization and severe environmental damage to their ancestral lands. This situation is largely due to the Nigerian federal government's exploitation of natural resources in collaboration with Western big oil companies in the Niger Delta [17]. Extensive oil extraction has led to environmental degradation, with pollution from spills and gas flaring causing significant health issues, such as respiratory diseases, cancers, and other issues, including increase in infant and maternal mortality rate in the region [18]. A 2019 study by Bruederlea and Hodler found that oil spills within 10 km of a mother's residence doubled neonatal mortality rates, even if the spill occurred 5 years before conception, significantly impacting child health in the Niger Delta [19]. The Ogoni have also been neglected in terms of healthcare and compensation, reflecting systemic discrimination. Recent reports continue to highlight ongoing pollution and unresolved health impacts, underscoring the persistent inequity and neglect driven by economic motives [20]. This shows intentional harming and neglect to save people's lives due to financial and political benefits, which clearly explains what health inequity is.

### 2.4 Root Causes of Health Inequalities, Disparities, and Inequities in Africa

Health inequalities, disparities, and inequities in Africa arise from a complex interaction of various factors. These root causes can be broadly categorized into elements related to socioeconomic factors, politics, economy, geography and environment, culture, and other underlying factors. Understanding these underlying causes is crucial for drafting and implementing effective interventions that can eliminate the observed differences in health outcomes among African populations. Addressing these issues holistically is essential to achieving equitable health for all.

Poverty and education differences are significant drivers of differences in health outcomes in Africa [21]. In many African countries, poverty limits access to healthcare services, as individuals in low‐income areas may be unable to afford special medical treatments or even basic healthcare [22]. Furthermore, as education plays a crucial role in health literacy, those with lower education levels often lack knowledge and skills regarding disease prevention and healthy practices and often delay to seek care for themselves and their families [23]. For instance, individuals with limited education might be less aware of the importance of vaccinations or prenatal care, impacting overall maternal and child health outcomes [24]. Additionally, low educational attainment affects economic opportunities, which in turn limits access to quality healthcare [8]. Addressing these issues requires targeted efforts to reduce poverty and improve educational opportunities to enhance health equity across the continent.

Political and governance issues also significantly contribute to health outcomes differences in Africa [25]. Inadequate funding and bad management of health systems often results in deficient infrastructure and a scarcity of medical supplies [26]. Furthermore, resources intended for vital health services are sometimes diverted by corruption and fraud and are used for personal interests instead of benefiting the public [27]. Additionally, health services and infrastructures are disrupted by political instability and conflict, as seen by the dire health crises in conflict areas, such as South Sudan [28]. Disparities are also worsened by unfair policy implementation where, for example, people in urban areas get better healthcare services and more resources than people living in rural areas [29]. Improving governance, eradicating corruption and fraud, and guaranteeing healthcare equity are necessary to address these problems.

Health disparities in Africa are also intervened by geographic and environmental factors. Urban–rural divides create disparities, as rural areas often lack access to good quality healthcare facilities, whereas people living in urban areas access them [30]. Geographic isolation further restricts access to healthcare as it can be extremely difficult for residents of isolated places to get to medical facilities [31]. Furthermore, treatment and access to necessary health services are further delayed by inadequate transportation infrastructure especially in rural areas [32]. On the environmental side, preventable waterborne diseases like cholera and malaria are more common in areas with inadequate sanitation and safe water supplies [33]. Additionally, climate change and extreme weather events sometimes cause droughts, which in turn cause food insecurity and malnutrition [34]. Improving infrastructure, addressing environmental issues, and putting effective climate adaption plans into action are all necessary to meet these challenges.

African cultural and social norms play a crucial role in shaping health disparities and inequities. Gender inequality often limits women's access to healthcare, whereas certain cultural traditions further restrict their ability to make decisions about reproductive health and seek medical care [35]. Discrimination and stigmatization impact marginalized groups, including LGBTQ+ people and those living with HIV/AIDS, making them feel unaccepted in the community, which often results in their exclusion from health care [36, 37, 38]. Harmful customs like female genital mutilation (FGM) are common in several African societies, which has unmeasurable effects on women's health and access to care [39]. Furthermore, in several African communities, the acceptance of modern medicine is also hampered by traditional beliefs, as some cultures favor traditional healers over medical experts [40]. These factors significantly contribute to the observed differences in health outcomes among different population groups in Africa.

In discussing health disparities and inequities in Africa, we cannot forget the role of external international factors. Global economic policies such as some unfair international trade agreements, debt loads, and economic measures enforced by global agencies have impacted healthcare in Africa [41, 42]. Additionally, priorities in global health frequently concentrate narrowly on particular diseases, such as HIV/AIDS or malaria, and this narrow emphasis often result in under prioritization of other concerning conditions that also need attention, creating disparities and inequities in different corners of health sector in African countries [43]. Furthermore, historical and colonial legacies also come into play; the colonialism and exploitation of African countries had a long‐lasting effect on social inequality, infrastructure, and health systems, creating disparities and inequity in the continent's health sector [44, 45, 46].

### 2.5 Recommendations to Tackle Health Inequality, Disparity, and Inequity in Africa

Understanding the concepts and root causes of health inequality, disparity, and inequity in Africa will not yield any change if no intervention is implemented. It is important for governments, African communities, international organizations, and stakeholders to collaboratively implement interventions that will address the differences in health outcomes among the African population. Implementing the following recommendations can help mitigate the observed differences in health outcomes in African countries (Table 1).

**TABLE 1:** Recommendations tackle health inequality, disparity and inequity in Africa.

Solution	Description	References
Ensuring education all	Ensuring universal access to education, particularly for girls and marginalized communities, to improve health literacy and economic opportunities	[47, 48]
Poverty reduction interventions	Implementing social protection programs to reduce poverty and increase access to healthcare services	[49]
Investing in building infrastructure	Developing transportation infrastructure to improve access to healthcare facilities, especially in geographically isolated regions	[32, 50, 51]
Funding health sector	Increasing national healthcare budgets to build better infrastructure, and ensuring the availability of essential healthcare to all	[52, 53]
Ensuring good governance	Establishing transparent governance structures to prevent corruption and fraud, ensuring equitable distribution of healthcare resources	[54, 55, 56]
Equal distribution of resources	Investing in healthcare facilities, staffing, and training in rural areas to reduce urban–rural health disparities	[57, 58]
Gender equality policy enforcement	Enforcing laws and policies that promote gender equality in access to healthcare and education, empowering women and girls	[59, 60, 61]
Ensuring inclusiveness in healthcare	Developing inclusive healthcare policies that address the needs of marginalized groups, including LGBTQ+ individuals and people living with HIV/AIDS, to reduce discrimination and stigma	[62, 63]
Trade and economic policy reform	Engaging in international conversations to renegotiate trade agreements and economic policies that negatively impact healthcare access in Africa	[52, 64]
Health inequality monitoring	Improving data collection and health information systems to monitor health disparities and inequities, enabling targeted interventions	[65, 66, 67, 68]

## 3 Conclusion

Health inequality, disparity, and inequity remain critical barriers to healthcare in Africa, stemming from socioeconomic challenges, geographic and environmental factors, political dynamics, cultural practices, and global influences. Tackling these disparities requires a multifaceted approach that addresses their root causes. Key interventions include improving education, reducing poverty, strengthening healthcare infrastructure, promoting gender equality, and ensuring good governance. Additionally, international collaboration and policy reforms are essential to bridging healthcare gaps. By implementing these measures, African nations can work toward equitable health outcomes, ensuring that healthcare services are accessible, affordable, and of high quality for all populations, particularly marginalized communities. Sustainable progress depends on coordinated efforts from governments, organizations, and stakeholders at all levels.

## Author Contributions

**Gabriel Ilerioluwa Oke**: conceptualization, methodology, validation, visualization, supervision, resources, project administration, writing – review and editing. **Olivier Sibomana**: conceptualization, writing – original draft, methodology, validation, visualization, writing – review and editing, data curation.

## Ethics Statement

The research conducted in this review did not involve any human or animal subjects and therefore did not require ethical approval. However, ethical guidelines were followed in the collection and analysis of data from secondary sources, ensuring that all information was accurately represented and properly cited.

## Conflicts of Interest

The authors declare no conflicts of interest.

## Data Availability

All data generated or analyzed during this study are included in this published article. Additional data supporting the findings of this study are available from the corresponding author upon reasonable request.
